# Supplementary material for: Assessment of Italian Population Awareness on One-Health, Zoonoses and the Mpox Vaccine: A Nationwide Cross-Sectional Study
Source: Vaccines (Basel). 2024 Mar 1;12(3):258. doi: 10.3390/vaccines12030258 (PMC10974339; doi:10.3390/vaccines12030258)
Supplement: Supplementary file 1 [file vaccines-12-00258-s001.zip › vaccines-2861131-supplementary.pdf]

Assessment of Italian Population Awareness on One-Health, Zoonoses and the Mpox Vaccine: a nationwide cross-sectional study

Fabrizio Bert, Giuseppina Lo Moro, Francesco Calabrese, Valentino Barattero, Alberto Peano, Giacomo
Scaioli and Roberta Siliquini

PART 1

Socio-demographic characteristics of the subjects

Gender: Male Female Other/Non-binary

Age: ___ years

Sexual orientation: Heterosexual Homosexual Bisexual Asexual Other

Nationality: Italian Other _________

Region of residence: _________

Do you live in a municipality of:

- ≤ 5,000 inhabitants
- 5,001 - 30,000 inhabitants
- 30,001 - 50,000 inhabitants
- 50,001 - 100,000 inhabitants
- 100,001 - 250,000 inhabitants
- 250,000 inhabitants

Marital status:

- Single
- Divorced
- Married
- Living together
- Widowed

What is the highest level of education you have completed?

- None
- Elementary school diploma
- Middle school diploma
- High school diploma
- Bachelor's degree
- Postgraduate degree

Occupation:

- Unemployed Student Employed Retired Homemaker Other ____

Do you work in the healthcare field? Yes No

Do you work with animals or are you studying for a profession that involves working with animals? Yes No

- If yes: What is your occupation/study program? Breeder Veterinarian Groomer Trainer Other _________

How would you describe the economic situation of your household?

- Excellent
- Good
- Poor
- Insufficient

Within your household (including yourself), are there:

- Children under 5 years old
- Pregnant women
- Elderly individuals Individuals with chronic illnesses
- Immunocompromised individuals
- None of the above

Do you have children? Yes No

Do you own pets? Yes No

If yes: Which ones? (multiple answers possible)

- Dogs
- Cats
- Rabbits
- Hamsters
- Birds
- Turtles
- Other: ___________

If No: Have you ever had pets?

If Yes: Which ones?

- Dogs
- Cats
- Rabbits
- Hamsters
- Birds
- Turtles
- Other: ___________

Do you own farm animals? Yes No

If yes: (multiple answers possible)

- Chickens
- Cows
- Goats
- Rabbits
- Pigs
- Horses
- Other: _______________

If No: Have you ever had farm animals?

If Yes: Which ones?

- Chickens
- Cows
- Goats
- Rabbits
- Pigs
- Horses
- Other: _______________

How would you rate your overall health?

- Very good
- Good
- Fairly good
- Poor
- Very poor

How often do you need assistance when reading instructions, brochures, or other material given to you by your doctor or pharmacist?

- Never
- Rarely
- Sometimes
- Often
- Always

Answer the following questions by assigning a score from 0 (never or almost never) to 3 (every day)

I feel nervous, tense, or anxious (from 0 to 3)

I cannot stop or control my worrying (from 0 to 3)

PART 2 One-Health

Do you believe humans can contract infections from:

- Animals
- Plants
- Pollution

- None of the above

Have you ever heard of One Health (OH)? Yes No

For each of the following topics, indicate whether you believe they are the main topics addressed by One Health: (True or False)

- Don't know Food safety
- Psychological well-being of workers
- Control of zoonoses
- Oncological screening (e.g., colon cancer screening)
- Neglected tropical diseases
- Antibiotic resistance
- Health related to environment and pollution
- Psychological interventions for behavior modification

In your opinion, are the following statements TRUE or FALSE:

- Human activities have created new opportunities for the spread of diseases.
- Water quality can affect human health.
- Antibiotic resistance is a problem only for people who regularly take antibiotics.
- Climate change cannot affect human health.
- Deforestation can affect human health.
- The use of antibiotics in animals can affect human health.

Have you ever heard of zoonoses? (Yes No)

In your opinion, zoonoses are:

- Diseases of animals that do not involve humans
- Diseases that humans transmit to animals
- Diseases transmitted from animals to humans exclusively through vectors (e.g., mosquitoes, ticks, fleas, lice)
- Diseases that can be transmitted from animals to humans
- Diseases transmitted from plants to animals

PART 2.2 ZOONOSES KNOWLEDGE

Read the definition: "Zoonoses are diseases caused by agents transmitted from animals to humans." (are the following statements TRUE or FALSE)

- Currently, less than 20% of emerging infectious diseases originate from animals.
- Zoonoses are transmitted from wild or farm animals but cannot be transmitted from domestic animals.
- Some zoonoses can be transmitted through contact with an animal's saliva.
- Washing hands after contact with animals helps protect against the risk of contracting a zoonosis.
- All zoonoses present with respiratory symptoms (e.g., cough, difficulty breathing).
- Some zoonoses can be transmitted through mosquitoes or ticks.

- Some zoonoses can be transmitted through water contaminated by feces from infected animals.
- Some zoonoses can be transmitted through foods produced by infected animals.
- Eating raw eggs puts me at risk of contracting a zoonosis.
- Zoonoses CANNOT be transmitted from contaminated objects.
- The trade of live wild animals can increase the likelihood of infectious diseases being transmitted to people.
- There are no zoonoses preventable with vaccines.
- Some zoonoses can be transmitted from person to person.

In your opinion, are the following diseases zoonoses? (True or False)

- COVID-19
- Chickenpox
- Monkeypox
- Salmonellosis
- Ebola

Part 2.3 Mpox vaccine

Do you think there is a vaccine that protects against monkeypox? Yes No Don't know

Part 2.4 Source of information

Where have you heard about One Health or zoonoses and found information about them? (multiple answers possible)

- I had never heard of them before this questionnaire
- Television: news programs
- Television: entertainment channels
- General practitioner (family doctor) Pharmacist Veterinarian
- Government websites Social media: official sources (e.g., Ministry, WHO)
- Social media: unofficial or alternative sources (Instagram or TikTok profiles, etc.) Scientific literature (studies and scientific articles)
- Newspapers (print or online, e.g., La Stampa, Repubblica, etc.)
- Friends/acquaintances School/university Radio
- Other

Through which channels would you prefer to receive information about zoonoses and epidemics? (multiple answers possible)

- I would not like to receive any information
- Short videos Infographics Synthetic illustrative sheets (brochures)
- List of references to various online sites containing certified information
- Training events organized by the Local Health Authority (ASL)
- Direct dialogue with my general practitioner (family doctor)
- Scientific dissemination television programs
- Television advertisements
- Social media (e.g., Facebook, Twitter, Instagram, TikTok)

- Dialogue with the veterinarian Updates during working hours
- Other

Conoscenze ed attitudini in merito a One-Health e Zoonosi: uno studio cross-sectional”

PARTE 1 caratteristiche socio demografiche dei soggetti

- Genere:
    - Uomo
    - Donna
    - Altro/Non Binario

- Età: ___ anni

- Orientamento sessuale:
    - Eterosessuale
    - Omosessuale
    - Bisessuale
    - Asessuale
    - Altro

- Nazionalità:
    - Italiana
    - Altro __________

- Regione di residenza: __________

- Lei vive in un comune di:
    - ≤ 5.000 abitanti
    - 5.001 - 30.000 abitanti
    - 30.001 - 50.000 abitanti
    - 50.001 - 100.000 abitanti
    - 100.001 - 250.000 abitanti
    - >250.000 abitanti

- Stato civile:
    - Single
    - Divorziato/a
    - Sposato/a
    - Convivente/a
    - Vedovo/a

- Qual è il titolo di studio più alto da Lei conseguito?
    - Nessuno
    - Licenza elementare
    - Licenza media
    - Diploma di scuola superiore
    - Laurea
    - Titolo post laurea

- Occupazione:
    - Disoccupato/a
    - Studente/essa
    - Lavoratore/trice
    - Pensionato/a
    - Casalingo/a
    - Altro _____

- Lavora in ambito sanitario?
    - Sì
    - No

- Lavora a contatto con gli animali o sta studiando per una professione per la quale è a contatto con animali?

- Sì
- No

- Se sì: Che lavoro fa/per quale professione sta studiando?
  - Allevatore/trice
  - Veterinario/a
  - Toelettatore/trice
  - Addestratore/trice
  - Altro _________

- Come definirebbe la situazione economica del suo nucleo familiare?
  - Ottima
  - Buona
  - Scarsa
  - Insufficiente

- All’interno del suo nucleo familiare sono presenti (incluso lei stesso): (è possibile indicare più di una risposta)
  - Bambini sotto i 5 anni
  - Donne incinta
  - Persone anziane
  - Persone con malattie croniche
  - Persone con immunodepressione
  - Non sono presenti persone con le suddette caratteristiche

- Ha figli/e? da Commodari et al. (6)
  - Sì
  - No

- Possiede animali domestici:
  - Sì
  - No

- Se sì: Quali? (possibile più di una risposta)
    - Cani
    - Gatti
    - Conigli
    - Criceti
    - Volatili
    - Tartarughe
    - Altro: ____________

- Se No: Ne ha mai avuti?

- Se Sì: Quali?
    - Cani
    - Gatti
    - Conigli
    - Criceti
    - Volatili
    - Tartarughe
    - Altro: ____________

- Possiede animali da allevamento
    - Sì
    - No

- Se sì: (possibile più di una risposta)
    - Galline
    - Mucche
    - Capre
    - Conigli
    - Maiali
    - Cavalli

- Altro: _______________

- Se No: Ha mai avuto animali da allevamento?

- Se Sì: Quali? Galline
    - Mucche
    - Capre
    - Conigli
    - Maiali
    - Cavalli
    - Altro: _______________

- Come va in generale la sua salute?
    - Molto buono
    - Buono
    - Abbastanza buono
    - Cattivo
    - Molto cattivo

- Quante volte ha bisogno di qualcuno che la aiuti quando legge istruzioni, opuscoli o altro materiale che le è stato consegnato dal proprio medico o farmacista?
    - Mai
    - Raramente
    - Qualche volta
    - Spesso
    - Sempre

- Risponda alle seguenti domande attribuendo un punteggio da 0 (mai o quasi mai) a 3 (tutti i giorni)

Mi sento nervosa/o, tesa/o o ansiosa/o	0	1	2	3
Non sono in grado di fermare o controllare le mie preoccupazioni	0	1	2	3

PARTE 2 One-Health

- Crede che gli esseri umani possano contrarre infezioni da:
  - Animali
  - Piante
  - Inquinamento
  - Nessuna delle precedenti

- Ha mai sentito parlare di One Health (OH):
  - o Sì
  - o No

- Per ciascuna delle seguenti tematiche, indichi se, secondo lei, si tratta delle tematiche principali di cui si occupa la One Health:

	Vero	Falso	Non so
Sicurezza alimentare			
Benessere psicologico dei lavoratori			
Controllo delle zoonosi			
Screening oncologico (ad esempio screening tumore colon)			
Malattie tropicali neglette			
Resistenza agli antibiotici			
Salute legata ad ambiente e inquinamento			

Interventi psicologici per la modifica dei comportamenti a rischio			

- Secondo lei, le seguenti affermazioni sono VERE o FALSE:

	Vero	Falso	Non so
Le attività umane hanno creato nuove opportunità per la diffusione di malattie.			
la qualità dell'acqua può influire sulla salute umana.			
La resistenza agli antibiotici è un problema solo per le persone che assumono antibiotici regolarmente.			
I cambiamenti climatici non possono influire sulla salute umana.			
Abbattere le foreste può influire sulla salute umana.			
Abbattere le foreste può influire sulla salute umana.			
L'uso di antibiotici negli animali può influire sulla salute umana.			

- Ha mai sentito parlare di zoonosi?
    - Sì
    - No
- Secondo lei, le zoonosi sono:
    - o Malattie degli animali che non coinvolgono l'uomo
    - o Malattie che l'uomo trasmette agli animali
    - o Malattie trasmesse dagli animali all'uomo esclusivamente tramite vettori (es: zanzare, zecche, pulci, pidocchi)
    - o Malattie che si possono trasmettere dagli animali all'uomo
    - o Malattie trasmesse dalle piante agli animali

PARTE 2.2

Legga la definizione: "Le zoonosi sono malattie causate da agenti trasmessi dagli animali all'uomo."

	VERO	FALSO	NON SO
Attualmente, meno del 20% delle malattie infettive emergenti ha origine dagli animali			
Le zoonosi si trasmettono da animali selvatici o da allevamento ma non si possono trasmettere da animali domestici.			
Alcune zoonosi possono essere trasmesse con contatto con la saliva di un animale.			
Lavare le mani dopo il contatto con animali aiuta a proteggersi dal rischio di contrarre			

una zoonosi.			
Tutte le zoonosi si manifestano con sintomi respiratori (ad esempio tosse, difficoltà respiratoria)			
Alcune zoonosi possono essere trasmesse tramite zanzare o zecche			
Alcune zoonosi possono essere trasmesse tramite acqua contaminata da feci di animali infetti			
Alcune zoonosi possono essere trasmesse tramite alimenti prodotti da animali infetti			
Mangiare uova crude mi mette a rischio di contrarre una zoonosi.			
Le zoonosi NON possono essere trasmesse da oggetti contaminati			
Il commercio di animali selvatici vivi può aumentare la probabilità che le malattie infettive si trasmettano alle persone.			

Non esistono zoonosi prevenibili con i vaccini.			
Alcune zoonosi possono trasmettersi da uomo a uomo			

CONOSCENZE ZOONOSI

- In generale, quanto è preoccupato/a di contrarre una zoonosi?
  - Molto
  - Abbastanza
  - Poco
  - Per nulla

- Nella vita di ogni giorno, quanto la preoccupa contrarre zoonosi da:

	Molto	Abbastanza	Poco	Per nulla
Animali				
Animali selvatici				
Animali domestici				
Altre persone				
Zanzare o zecche				

- Secondo lei, le seguenti malattie sono zoonosi?

	Sì	No	Non lo so
COVID-19			
Varicella			
Vaiolo di scimmie			
Salmonellosi			
Ebola			

- Quante dosi della vaccinazione anti-COVID-19 ha fatto?

- Secondo lei, esiste un vaccino che protegga contro il vaiolo delle scimmie?
  - Sì
  - No
  - Non so

- In che contesto ha sentito parlare di One Health o zoonosi e trovato informazioni su di esse? (è possibile inserire più di una risposta)
  - Non ne avevo mai sentito parlare prima di questo questionario
  - Televisione: telegiornali
  - Televisione: canali di intrattenimento
  - Il proprio medico di Medicina Generale (medico di famiglia)
  - Farmacista
  - Veterinario
  - Siti ministeriali
  - Social media: fonti ufficiali (esempio: Ministero, OMS)
  - Social media: fonti non ufficiali o alternative (Profili instagram o Tiktok, …)
  - Letteratura scientifica (studi e articoli scientifici)
  - Giornali cartacei o online (es La Stampa, Repubblica, etc.)
  - Amici/conoscenti
  - Scuola/università
  - Radio
  - Altro:

- Attraverso quali modalità preferirebbe ricevere informazioni riguardo zoonosi ed epidemie? (è possibile inserire più di una risposta)
  - Non vorrei ricevere nessuna informazione

- Brevi video
- Infografiche
- Fogli illustrativi sintetici (brochure)
- Elenco di riferimenti a vari siti online contenenti informazioni certificate
- Eventi formativi organizzato dall’ASL
- Dialogo diretto con il proprio medico di medicina generale (medico di famiglia)
- Programma televisivo di divulgazione scientifica
- Pubblicità televisiva
- Social media (es. Facebook, Twitter, Instagram, TikTok)
- Dialogo con il veterinario
- Aggiornamenti durante l’orario di lavoro
- Altro:

Grazie per la collaborazione!
